# Characterization and Determination of the Antibacterial Activity of *Baccharis dracunculifolia* Essential-Oil Nanoemulsions

**DOI:** 10.3390/antibiotics12121677

**Published:** 2023-11-29

**Authors:** Erika da Silva Monteiro, Franklyn Santos da Silva, Karolina Oliveira Gomes, Bruno Alcântara do Prado, Rebeca Dias dos Santos, Claudio Augusto Gomes da Camara, Marcilio Martins de Moraes, Izabel Cristina Rodrigues da Silva, Vinicius Teixeira de Macêdo, Guilherme Martins Gelfuso, Lívia Cristina Lira de Sá Barreto, Daniela Castilho Orsi

**Affiliations:** 1 Laboratory of Quality Control, University of Brasília, Brasília 72220-900, DF, Brazil; erikamonteirounb@gmail.com (E.d.S.M.); franklyn.silva@aluno.unb.br (F.S.d.S.); gomes.karolina@aluno.unb.br (K.O.G.); prado.bruno@aluno.unb.br (B.A.d.P.); santos.rebeca@aluno.unb.br (R.D.d.S.); belbiomedica@gmail.com (I.C.R.d.S.); vinicius.macedo@escs.edu.br (V.T.d.M.); 2 Department of Chemistry, Federal Rural University of Pernambuco, Recife 52171-900, PE, Brazil; claudio.camara@ufrpe.br (C.A.G.d.C.); marcilio.moraes@ufrpe.br (M.M.d.M.); 3 Laboratory of Food, Drugs, and Cosmetics, University of Brasília, Brasília 70910-900, DF, Brazil; gmgelfuso@unb.br (G.M.G.); liviabarreto@unb.br (L.C.L.d.S.B.)

**Keywords:** essential oil, nanoemulsion, chemical composition, antimicrobial activity, minimal inhibitory concentration, minimal bactericidal concentration

## Abstract

The aim of this study was to evaluate the antibacterial activity of nanoemulsions of *Baccharis dracunculifolia* essential oil. The volatile compounds of the essential oil were identified using gas chromatography–mass spectrometry. The properties of the nanoemulsions (droplet size, polydispersity index, pH, and electrical conductivity) were determined. The antibacterial activities of the essential oil and its nanoemulsions were evaluated using MIC, MBC, and disk diffusion. The microorganisms used were: Gram-positive bacteria (*Staphylococcus aureus* ATCC 25923, *Bacillus cereus* ATCC 14579, *Streptococcus mutans* ATCC 25175, and *Enterococcus faecalis* ATCC 29212) and Gram-negative bacteria (*Pseudomonas aeruginosa* ATCC 27853, *Klebsiella pneumoniae* ATCC BAA-1706, *Salmonella enterica* ATCC 14028, and *Escherichia coli* ATCC 25922). The major volatile compounds of the *B. dracunculifolia* essential oil were limonene (19.36%), (*E*)-nerolidol (12.75%), bicyclogermacrene (10.76%), and β-pinene (9.60%). The nanoemulsions had a mean droplet size between 13.14 and 56.84 nm. The nanoemulsions presented lower and statistically significant MIC values compared to the essential oil, indicating enhancement of the bacteriostatic action. The disk diffusion method showed that both the nanoemulsions and the essential oil presented inhibition zones only for Gram-positive bacteria, while there were no results against Gram-negative bacteria, indicating that *B. dracunculifolia* essential oil has a better antimicrobial effect on Gram-positive microorganisms.

## 1. Introduction

The resistance to antimicrobials has been identified as one of the major problems for human and animal health in the 21st century. One of the major reasons for accelerated antibiotic resistance is their abusive and irrational use [1]. Another worrying factor is that, from 1980, there has been a decline in the approval of new antibiotics, as well as in the discovery of new classes [2,3], and, for this reason, new antibiotic drugs are urgently needed. In this scenario, plant-derived products such as essential oils represent a good source of bioactive compounds that act as antimicrobials. In fact, several antibiotic drugs have been obtained from different plant species, and most of the available antibiotics have been obtained from compounds of natural origin [4,5,6].

*Baccharis dracunculifolia* DC (Asteraceae) is a perennial bush tree that reaches a height of 2–3 m; in Brazil, it is commonly called “alecrim-do-campo” and “vassourinha”. It occurs naturally in the Brazilian biomes of Atlantic Rainforest, Pampas, and Cerrado [7]. The species *Baccharis dracunculifolia*, *Araucaria angustifolia*, and *Eucalyptus citriodora* are cited as the most-used plant sources for the production of propolis by bees in Brazil [8]. Both propolis and the essential oil obtained from *B. dracunculifolia* leaves have aroused great interest in the pharmaceutical industry, because they contain bioactive compounds that have important pharmacological properties, such as antibacterial and antifungal activities [7,9].

Studies have identified sesquiterpenes as the major components of *B. dracunculifolia* essential oil. Cazella et al. [7] found 83.7% of sesquiterpenes and 9.6% of monoterpenes in the essential oil of *B. dracunculifolia*, and the main compounds were spathulenol (27.4%) and trans-nerolidol (23.1%) (oxygenated sesquiterpenes). Salazar et al. [10] reported 78.9% sesquiterpenes and 16.5% monoterpenes in the essential oil of *B. dracunculifolia* from the Atlantic Forest in the State of Paraná, Brazil, and the main constituent was the sesquiterpene germacrene D (18.4%). The presence of terpenes in essential oils has been associated with their antimicrobial action, as these compounds cause cell membrane rupture and increased permeability, leading to cell death [11,12].

Several studies have reported the antimicrobial properties of *B. dracunculifolia* essential oil. Cazella et al. [7] found that the essential oil showed bactericidal activities against *Staphylococcus aureus*, *Bacillus cereus*, and *Pseudomonas aeruginosa*. Salazar et al. [10] observed that the essential oil synergistically modulated the antimicrobials norfloxacin and gentamicin against *P. aeruginosa* and *E. coli*, with a reduction in the minimum inhibitory concentration. Luchesi et al. [9] reported that the essential oil led to a significant inhibition of the growth of the phytopathogenic fungus Fusarium *graminearum*. Pedroti et al. [13] found antifungal activity of the essential oil against *Botrytis cinerea* and *Colletotrichum acutatum*. And Brandenburg et al. [14] reported another biological effect of *B. dracunculifolia* essential oil, namely, that it has an anti-inflammatory activity in skin inflammation.

However, the low aqueous solubility, volatility, and sensitivity to the environment displayed by essential oils are characteristics that make it difficult to develop suitable pharmaceutical formulations. Therefore, an alternative mechanism for increasing the stability and ensuring the effectiveness of essential oils could be the production of nanoemulsions [15,16]. Essential oils can be encapsulated in delivery systems to optimize the oils’ solubility, stability, and controlled release. The formulation of nanoemulsions increases the surface-to-volume ratio, resulting in better effective absorption through cells, in addition to regulated release and targeting of bioactive substances at the sites of action [4,17,18].

Nanoemulsions are systems with nanometer-sized droplets stabilized by emulsifiers, thus consisting of a colloidal system of two immiscible liquids which are dispersed in a continuous phase that can be aqueous (oil in water) or oily (water in oil) [19]. Due to their small droplet-size, nanoemulsions have high levels of stability compared to conventional emulsions, and typically require a low concentration of surfactants for their formulation, which makes them a better option than microemulsions for use as a carrier system [20]. Additionally, nanoemulsions, due to their smaller particle sizes compared to microemulsions, can more easily penetrate into the cytoplasmic membranes of microorganisms, increasing the antimicrobial activity of essential oils and allowing reduction in the doses needed to show antimicrobial activity [21].

Although there are significant data available on the application of *B. dracunculifolia* essential oil as an antimicrobial agent, no comparison between the antibacterial properties of the essential oil and its nanoemulsions has been documented. Thus, the aim of this study was to evaluate the antibacterial activity of these nanoemulsions and compare the results with those of the *B. dracunculifolia* essential oil.

## 2. Results and Discussion

### 2.1. Identification of Major Volatile Compounds of Baccharis dracunculifolia Essential Oil

The analysis of the chemical composition of the *B. dracunculifolia* essential oil allowed the identification of 27 volatile compounds, representing 99.56% of the total oil (Table 1).

The components detected in greater quantities were limonene (20.00%), (*E*)-nerolidol (13.01%), bicyclogermacrene (10.76%), and β-pinene (9.60%). The results of this work were similar to those of previous studies. Brandenburg et al. [14] reported that the main compounds of *B. dracunculifolia* essential oil were limonene (6.76%), β-caryophyllene (8.44%), bicyclogermacrene (14.18%), and nerolidol (8.02%). Salazar et al. [10] showed that the major compounds of *B. dracunculifolia* essential oil were germacrene D (18.4%), (*E*)-nerolidol (14.0%), spathulenol (11%), β-pinene (9.5%), and bicyclogermacrene (8.4%). Luchesi et al. [9] reported nerolidol (17.58%), γ-elemene (15.06%), D-limonene (10.54%), caryophyllene (9.76%), and β-pinene (9.57%) as major compounds of *B. dracunculifolia* essential oil. And Pedrotti et al. [13] obtained β-pinene (18.01%), spathulenol (13.43%), and limonene (10.11%) as major compounds of *B. dracunculifolia* essential oil.

### 2.2. Characterization of Nanoemulsions

The nanoemulsions of *B. dracunculifolia* essential oil had a mean droplet size between 13.14 and 56.84 nm (Table 2). According to the literature, nanoemulsions must have droplets with a diameter of 100 nm or smaller [22,23]. A smaller droplet size of the nanoemulsions helps suppress coalescence and droplet precipitation, thereby minimizing nanoemulsion breakdown [24].

Periasamy et al. [25] reported similar results, in which nanoemulsions of cumin essential oil (*Nigella sativa* L.) showed droplets with sizes between 20 and 50 nm. The small size of the nanoparticles allows them to pass through different biological barriers to deliver drugs at various levels. In fact, the smaller the particle size, the greater the specific surface, reactivity, and bioavailability of the entrapped drug, resulting in enhanced bioactive functionalities, such as antimicrobial efficacy [1].

Anwer et al. [26] observed that nanoemulsions of clove essential oil (*Syzygium aromaticum* L.) showed a wider variation in droplet size (29.1–136.9 nm) among the different formulations tested, with formulations containing the highest concentrations of oil-phase having larger droplet sizes. In the present study, similar results were observed, in which the nanoemulsions formulated with the highest concentrations of essential oil of *B. dracunculifolia* showed larger droplet sizes compared to the nanoemulsions formulations with lower concentrations of essential oil.

The sizes of the droplets may vary in nanoemulsions due to the differences in the procedures used, given that the size of the particles tends to decrease with the increase of the sonication time or as determined by the pressure used in the homogenization, due to the composition of each essential oil, as well as due to the concentration of surfactant used, since a higher concentration of surfactants in the nanoemulsions can reduce the diameter of the particles [24,27].

The proportion of surfactant in the nanoemulsion can be verified by the value of the oil–surfactant ratio (SOR), which is below 1 in nanoemulsions [28,29]. In the present study, the SOR values varied between 0.50 and 0.89. It was also noted that the higher the SOR value, the smaller the particle size. This occurs since an increase in the proportion of surfactant can lead to adsorption at the interface of the droplets, forming a protective layer, and thus avoiding coalescence, that is, the process by which two or more droplets merge to form a larger droplet [28].

The polydispersion index (PDI) is a representation of the distribution of population sizes within a sample. The value of the PDI ranges from 0.0 (for a perfectly uniform sample with respect to particle size) to 1.0 (for a highly polydisperse sample with populations of various particle sizes). In drug delivery applications using lipid-based carriers, a PDI of 0.3 and below indicates a homogenous population, and PDI values bigger than 0.7 indicate that the sample has a very broad particle-size distribution [30].

In the present study, the PDI values ranged from 0.12 to 0.30. Periasamy et al. [25] reported that nanoemulsions of cumin essential oil (*Nigella sativa* L.) showed PDIs from 0.11 to 0.22. And the thyme essential oil (*Thymus vulgaris*) nanoemulsion showed a PDI of 0.16 [31].

The Zeta potential is used to measure the surface charge of nanoparticles when placed in a liquid [32]. Zeta potential is determined by measuring the velocity of charged particles moving towards the electrode through the sample solution in the presence of an external electric field and can range from +100 to −100 mV. The measurement of the Zeta potential is used to predict the stability of the dispersion, and its value depends on the physical–chemical properties, presence of electrolytes, and adsorption capacity of the nanoparticles [33].

In the present work, negative surface charges (−4.14 to −23.03 mV) were observed. Anwer et al. [26] observed Zeta potential values from −48.6 to −31.4 mV for clove essential-oil (*Syzygium aromaticum* L.) nanoemulsions. Seibert et al. [34] reported that a lemongrass essential-oil (*Cymbopogon densiflorus*) nanoemulsion showed a value of −8.98 mV for the Zeta potential and remained stable in the accelerated stability study, as evaluated by centrifugation and thermal stress tests at 24 h, and then again at 7, 14, 21, and 28 days after the preparation of the formulations.

The conductivity value determines the number of free ions in the system, therefore indicating the nanoemulsion’s ability to conduct electricity. Studies report that oil/water systems have higher conductivity measurements than water/oil systems because the external phase is water [35]. There are no established values in the literature for the conductivity of nanoemulsions; however, a higher value of nanoemulsion conductivity demonstrates greater water content, as it provides more space for ion movements [36]. The conductivity values presented in the present work varied between 105.70 and 120.80 µS/cm, and thus the nanoemulsions were classified as oil/water.

Seibert et al. [34] observed that the nanoemulsion of lemongrass essential oil (*Cymbopogon densiflorus*) presented a conductivity of 159.86 µS/cm as an oil/water type nanoemulsion. El-Ekiaby et al. [37] also obtained a basil essential-oil (*Ocimum basilicum* L.) nanoemulsion with a conductivity of 203.5 µS/m, being classified as oil/water. Moradi and Barati [36] observed a higher conductivity value of up to 777 µS/cm in nanoemulsions prepared with thyme (*Thymus vulgaris* L.) and rosemary (*Rosmarinus officinalis* L.) essential oils, indicating a water/oil system.

In the present work, the nanoemulsions presented pH values between 6.73 and 7.55. Similar values were observed by Moradi and Barati [36], where the pH of nanoemulsions with essential oils of thyme (*Thymus vulgaris* L.) and rosemary (*Rosmarinus officinalis* L.) presented pH values of 6.75 to 7.93, without variations for two months, indicating stable formulations. Narawi et al. [38] obtained nutmeg essential-oil (*Myristica fragrans*) nanoemulsions with pH values between 5.60 and 5.30, indicating suitable values for topical administration to the skin, since the skin’s pH is slightly acidic, in the range of 6.00.

### 2.3. Antibacterial Activity of Nanoemulsions and B. dracunculifolia Essential Oil

The *B. dracunculifolia* essential oil presented MIC values of 10 to 15 mg/mL and MBC values of 15 to 20 mg/mL, and the *B. dracunculifolia* nanoemulsions presented MIC values of 2.5 to 7.5 mg/mL and MBC values from 10 to >20 mg/mL (Table 3). The nanoemulsions presented lower and statistically significant MIC values compared to the essential oil, indicating enhancement of the bacteriostatic action.

Similar results were reported by Yazgan et al. [39], who observed that the nanoemulsion of lemon essential oil (*Citrus limonum*) showed a better inhibitory effect on *S. aureus* and *K. pneumoniae* (MIC values of 3.13–6.25 mg/mL) compared with lemon essential oil (MIC value of 12.50 mg/mL). Hassanshahian et al. [40] observed that the nanoemulsion from camelthorn-bush essential oil (*Alhagi maurorum*) inhibited the growth of all bacteria tested (*S. aureus*, *B. cereus*, *P. aeruginosa*, *K. pneumoniae*, *E. coli,* and *Acinetobacter baumannii*) at a lower concentration (MIC values of 1.75–12.50 mg/mL) compared to free essential oil (MIC values of 3.25–25.00 mg/mL).

As for MBC values, nanoemulsions showed results with lower and statistically significant concentrations only for *S. aureus* and *S. mutans*. The MBC values of the *B. dracunculifolia* nanoemulsions ranged between 10 and 20 mg/mL for Gram-positive bacteria and were >20 mg/mL for Gram-negative bacteria, showing that Gram-negative bacteria were less sensitive to the bactericidal action of nanoemulsions than were Gram-positive bacteria.

Similar results were reported by Yazgan et al. [41], who observed that nanoemulsion of sage essential oil (*Salvia officinalis*) showed a better inhibitory effect on *E. faecalis* (Gram-positive bacteria), with MIC values of 12.50 mg/mL, compared to sage essential oil (MIC > 25.00 mg/mL). On the other hand, for *Serratia liquefaciens* (Gram-negative bacteria), the sage essential oil showed a lower concentration (MIC of 6.25 mg/mL) compared to the nanoemulsion (MIC of 12.50 mg/mL).

Rosato et al. [5] observed that for Gram-positive bacteria, the MIC values of the *Carlina acaulis* essential-oil nanoemulsions resulted in higher values (between 7.5 and 60 mg/mL) in relation to essential oil (between 0.68 and 2.9 mg/mL), a result probably obtained due to the low concentration of essential oil in the nanoemulsion system. The tests showed a complete inefficacy of *Carlina acaulis* essential oil against the Gram-negative species tested.

Shahabi et al. [42] observed that the MIC and MBC values of nanoemulsion and essential oil of Shirazi thyme (*Zataria multiflora* Boiss) against *Salmonella* Typhimurium were 5 mg/mL, and against *Listeria monocytogenes* were 2.5 mg/mL. According to the authors, converting the essential oil to nanoemulsion does not always greatly improve its antibacterial activity.

The differences in the MIQ and MBC values of the nanoemulsions may be related to factors such as the form of drug release from the nanoemulsions, which in in vitro studies show an initial rapid release followed by a slow release. Thus, the release of controlled doses of essential oil over 24 h leads to the control of bacterial growth, causing a better bacteriostatic effect compared to that of free essential oil [43]. And it should also be considered that compounds considered bacteriostatic act at lower concentrations, while those considered bactericidal require higher concentrations [44].

Also, the differences in antimicrobial activity between nanoemulsions are related to several factors, such as the characteristics of each essential oil. Essential oils can be extracted from a wide variety of plants which present complex combinations of volatile secondary metabolites, leading to a variety of biological properties [45]. Another important consideration is the formulation of the nanoemulsion, which depends on the concentrations of oil/water/surfactant and the emulsification process used, and may have different characteristics, and thus act on bacteria through different mechanisms of action [46]. Thus, the different physicochemical properties of the formulations can alter the antimicrobial properties of nanoemulsions [47].

Table 4 shows the results of antimicrobial activity obtained by the disk diffusion method for the nanoemulsions and the essential oil of *B. dracunculifolia*. Both the nanoemulsions and the essential oil showed inhibition zones only for Gram-positive species (*S. aureus*, *B. cereus* and *S. mutans*), while there were no results for Gram-negative species (*P. aeruginosa*, *K. pneumoniae*, *S. enterica* and *E. coli*).

Similar results were presented by Özogul et al. [47], in which the disk diffusion method was used, and the nanoemulsion of laurel essential oil (*Laurus nobilis*) was more effective against Gram-positive bacteria (*S. aureus*, 15.00 mm; and *E. faecalis*, 11.25 mm) than against Gram-negative bacteria (*Salmonella* Paratyphi, no inhibition zone; and *Klebsiella pneumoniae*, 7.75 mm). Hassanshahian et al. [40] observed, using the disk diffusion method, that essential oil from camelthorn bush (*Alhagi maurorum*) and its nanoemulsion presented higher inhibition-zone values for Gram-positive bacteria, with 13–26 mm for *S. aureus* and 11–24 mm for *B. cereus*, and lower values for Gram-negative bacteria, with 6–15 mm for *P. aeruginosa* and 7–18 mm for *E. coli*.

In general, essential oils exhibit a more pronounced antimicrobial effect on Gram-positive bacteria. Gram-negative bacteria possess an outer membrane rich in lipopolysaccharides which limit the diffusion of hydrophobic compounds through it, thereby hindering the entry of the various antimicrobial components found in essential oils. On the other hand, Gram-positive bacteria do not have this outer membrane rich in lipopolysaccharides, which allows the hydrophobic constituents of essential oils to infiltrate the cell membrane, leading to increased ionic permeability, leakage of intracellular contents, and eventual cell death. However, essential oils can still exert an antimicrobial activity on Gram-negative bacteria, since porin proteins within the outer membrane form channels of sufficient size to permit the passage of small-molecular-mass compounds [48].

*B. dracunculifolia* essential-oil nanoemulsions have the potential to be applied in combination with conventional antimicrobials in future studies. Several studies have reported synergistic effects resulting from combinations of essential oils and traditional antimicrobials as new strategies to combat multidrug-resistant bacteria [49]. However, the use of combination therapies to combat infections can be challenging, especially when the phytochemicals and antimicrobial agents have low aqueous solubility and a limited ability to penetrate the cytoplasmic membranes of microorganisms, limiting their potential in treating infections. Nanoemulsions provide versatile platforms for addressing the challenges involved in using hydrophobic antimicrobials, including essential oils, to treat infections [50]. Nabawy et al. [50] reported a synergistic combination therapy approach for the treatment of wound biofilms based on nanoemulsions loaded with eugenol and the hydrophobic antimicrobial agent triclosan. The nanoemulsions demonstrated synergistic eradication of bacterial biofilms and biofilm-derived persister cells, with minimal toxicity to mammalian cells. Significantly, the combination of eugenol and triclosan exhibited better antimicrobial activity compared to triclosan alone.

## 3. Material and Methods

### 3.1. Baccharis Dracunculifolia Essential Oil

The essential oil used in the tests was purchased from a commercial establishment located in São Paulo, Brazil. According to the producer’s specification, the *B. dracunculifolia* essential oil was extracted from the leaves using a steam distillation technique.

### 3.2. Identification of Major Volatile Compounds of Baccharis dracunculifolia Essential Oil

The essential oil was subjected to chemical characterization using gas chromatography–mass spectrometry (GC-MS). Qualitative GC-MS analysis was conducted on a Shimadzu Chromatograph system (model QP2010 SE Plus, Shimadzu, Kyoto, Japan) equipped with a mass selective detector. The mass spectrometer operated at electron impact (EI) mode with an energy of 70 eV, employing a scan interval of 0.5 s and detecting fragments within the mass range of 40 to 550 Da. The analysis was performed on the same column and under the same temperature program as the GC-FID experiments, with the following parameters: helium was used as the carrier gas, flowing at a rate of 1 mL min^−1^ in split mode (1:30). A volume of 1 µL from a diluted solution (1/100) of the oil in *n*-hexane was injected using an auto injector AOC-20i. The components of the essential oil were identified based on GC-MS retention indices with reference to a homologous series of C8-C40 *n*-alkanes calculated using the Van der Dool and Kratz [51] equation and by computer matching against the mass spectral library of the GC-MS data system (NIST and WILEY 14th), as well as co-injection with authentic standards and consultation of other published mass spectra [52].

Area percentages were obtained from the GC-FID response without the use of an internal standard or correction factors. Quantitative gas chromatography (GC) analysis was performed utilizing a PerkinElmer Clarus 500 GC (Waltham, MA, USA) instrument, which was equipped with a flame ionization detector (FID) and employed a non-polar DB-5 fused silica capillary column (30 m × 0.25 mm × 0.25 μm). The temperature within the oven was programmed to increase from 60 to 240 °C at a rate of 3 °C per minute. Both the injector and detector temperatures were set at 260 °C. Hydrogen served as the carrier gas, with a flow rate of 1 mL per minute in split mode (1:30). The injection volume comprised 1.0 µL of a diluted solution (1/100) of the oil in n-hexane. Quantification of individual compounds was achieved by calculating their relative percentages in the chromatograms based on the GC-FID peak areas, following the elution order of the DB-5 column.

### 3.3. Preparation of B. dracunculifolia Nanoemulsions

The nanoemulsions were prepared using the essential oil of *B. dracunculifolia* (2.5–20.0% *w*/*w*), Tween 80 as the surfactant, and ultrapure water (Supplementary Materials Figure S1). The surfactant was maintained at a fixed concentration of 20% (*w*/*w*) and the ultrapure water was adjusted for each of the nanoemulsions, as shown in Table 5. The mixture was homogenized by the use of an ultrasonic processor (Vibra-Cell VC 750, Sonics & Materials, Inc., Newtown, CT, USA). The formulations were sonicated for 10 min; each cycle consisted of 30 s on and 30 s off pulses, as described by Prakash et al. [53]. During this process, the temperature was controlled by the use of ice around the beaker.

### 3.4. Characterization of Nanoemulsions

The droplet size, polydispersity index, and zeta potential of the nanoemulsions were measured using the Zetasizer Nano ZS (Malvern Panalytical, Malvern, UK). The pH was measured by a calibrated digital pHmeter (MPA-210 Model, MS-Tecnopon, São Paulo, Brazil), with the direct immersion of the electrode in the nanoemulsions at 25 ± 1 °C. The electrical conductivity of the nanoemulsions was measured by a digital conductivity meter (Instrutherm CD 820; São Paulo, Brazil).

### 3.5. Determination of Minimal Inhibitory Concentration (MIC) and Minimal Bactericidal Concentration (MBC) of B. dracunculifolia Essential Oil and Nanoemulsions

For the determination of the MIC and MBC for the disk diffusion of the essential oil of *B. dracunculifolia* and the nanoemulsions, the following microorganisms were used: Gram-positive bacteria (*Staphylococcus aureus* ATCC 25923, *Bacillus cereus* ATCC 14579, *Streptococcus mutans* ATCC 25175, and *Enterococcus faecalis* ATCC 29212) and Gram-negative bacteria (*Pseudomonas aeruginosa* ATCC 27853, *Klebsiella pneumoniae* ATCC BAA-1706, *Salmonella enterica* ATCC 14028, and *Escherichia coli* ATCC 25922). The bacterial inoculum was prepared using direct suspension of microbial growth in Müller–Hinton broth, with turbidity adjusted between 0.08 and 0.10 on a spectrophotometer at 625 nm (equivalent to the McFarland standard 0.5, and 1.0 × 10^8^ CFU/mL). Then, the bacterial inoculum was diluted in Müller–Hinton broth at a ratio of 1:150, resulting in a concentration of 1.0 × 10^6^ CFU/mL.

The MIC and MBC were performed according to the Clinical and Laboratories Standards Institute method [54], with adaptations. The essential oil was diluted in Müller–Hinton broth, and concentrations varied from 20 to 3 mg/mL; the nanoemulsions were tested in the concentrations of 20.0 to 2.5 mg/mL. Then, 0.1 mL portions of different concentrations of essential oils and nanoemulsions were distributed in a 96-well microtiter plate containing 0.1 mL volumes of bacterial inoculum suspensions. The positive control (demonstrating bacterial growth) consisted of 0.1 mL of the bacterial inoculum and 0.1 mL of Müller–Hinton broth. The negative control (indicating inhibition of bacterial growth) was composed of 0.1 mL of the essential oil and nanoemulsions, combined with 0.1 mL of Müller–Hinton broth. The controls were incubated for 24 h at 37 °C. For MBC tests, 100 μL aliquots from each incubated sample were spread onto Müller–Hinton agar plates and subsequently incubated for 18–24 h at 37 °C. The MBC was determined as the lowest concentration at which no visible bacterial growth was observed on the agar (Figure S2). For MIC tests, bacterial growth was assessed by adding 20 µL of a 0.01% *w/v* resazurin dye to each well. Resazurin is a redox indicator employed to evaluate cell viability. Initially non-fluorescent and blue, it turns pink and fluorescent when reduced to resorufin by oxidoreductases within viable cells [55]. Hence, the MIC was defined as the lowest concentration at which there was no change in color (Figure S3).

### 3.6. Antimicrobial Activity of B. dracunculifolia Essential Oil and Nanoemulsions Determined by the Disk Diffusion Method

The disk diffusion method was performed in accordance with the Clinical and Laboratory Standards Institute method [56]. To perform the disk diffusion test, the microbial inoculum was seeded, with the aid of a sterile swab, on the surface of a Müller–Hinton agar plate until a uniform smear was obtained. After the inoculum was dried, filter paper discs, 6 mm in diameter, impregnated with 10 μL of *B. dracunculifolia* essential oil and nanoemulsions, were applied. The results were obtained after 24 h of incubation at 37 °C by measuring the inhibition zones in millimeters (Figures S4 and S5).

### 3.7. Statistical Analysis

All experiments were performed as triplicates. Data were analyzed considering the analysis of variance and using STATISTICA^®^ software version 10.0. A *p* < 0.05 was statistically significant.

## 4. Conclusions

In the current research effort, it was observed that the nanoemulsions of *B. dracunculifolia* essential oil presented lower and statistically significant MIC values compared to the essential oil, indicating enhancement of the bacteriostatic action. However, for MBC values, nanoemulsions showed results with lower and statistically significant concentrations only for *S. aureus* and *S. mutans*, and Gram-negative bacteria were less sensitive to the bactericidal action of nanoemulsions than were Gram-positive bacteria. The disk diffusion method showed that both the nanoemulsions and the essential oil presented inhibition zones only for Gram-positive bacteria, while there were no results against Gram-negative bacteria, indicating that *B. dracunculifolia* essential oil has a better antimicrobial effect on Gram-positive microorganisms. In conclusion, *B. dracunculifolia* essential oil and its nanoemulsions have the potential to be used as natural antimicrobial agents against pathogenic bacteria in human and veterinary medicine.

## Figures and Tables

**Table 1:** Chemical composition (%) of *Baccharis dracunculifolia* essential oil.

	Compounds	Composition (%)	Classification
1	Tricyclene	0.35	Monoterpene
2	*α*-Pinene	6.00	Monoterpene
3	Sabinene	0.40	Monoterpene
4	*β*-Pinene	9.60	Monoterpene
5	Myrcene	2.27	Monoterpene
6	Limonene	20.00	Monoterpene
7	*γ*-Terpinene	0.53	Monoterpene
8	Terpinolene	0.27	Monoterpene
9	*α*-Cubebene	0.41	Sesquiterpene
10	*β*-Elemene	0.49	Sesquiterpene
11	*β*-caryophyllene	6.43	Sesquiterpene
12	Aromadendrene	2.96	Sesquiterpene
13	*α*-Humulene	1.51	Sesquiterpene
14	*β*-Santalene	1.50	Sesquiterpene
15	Cabreuva oxide C	1.20	Sesquiterpene
16	*γ*-Gurjunene	6.18	Sesquiterpene
17	*γ*-Muurolene	0.41	Sesquiterpene
18	Bicyclogermacrene	10.76	Sesquiterpene
19	*α*-Muurolene	1.30	Sesquiterpene
20	*Trans*-*β*-guaiene	0.60	Sesquiterpene
21	*γ*-Cadinene	1.41	Sesquiterpene
22	*α*-Cadinene	5.42	Sesquiterpene
23	(*E*)-nerolidol	13.01	Sesquiterpene
24	Spathulenol	4.30	Sesquiterpene
25	*β*-Copaen-4-*α*-ol	1.53	Sesquiterpene
26	1-*epi*-Cubebol	0.33	Sesquiterpene
27	7-*epi*-*α*-Eudesmol	0.39	Sesquiterpene
Total		99.56	
Monoterpenes		39.42	
Sesquiterpenes		60.14	

**Table 2:** Characterization of nanoemulsions of *B. dracunculifolia* essential oil.

Nanoemulsions	Size (nm)	SOR	PDI	Zeta Potential (−mV)	Conductivity (µS/cm)	pH
F1	56.84 ± 0.80	0.50	0.30 ± 0.02	4.14 ± 0.91	105.70 ± 0.00	7.55 ± 0.00
F2	42.89 ± 0.43	0.57	0.35 ± 0.07	12.77 ± 2.10	110.80 ± 0.00	7.01 ± 0.00
F3	20.45 ± 0.27	0.67	0.18 ± 0.01	9.82 ± 0.99	116.00 ± 0.00	6.95 ± 0.00
F4	16.62 ± 0.15	0.73	0.20 ± 0.01	23.03 ± 2.15	119.60 ± 0.00	6.92 ± 0.00
F5	15.46 ± 3.88	0.80	0.20 ± 0.11	22.77 ± 1.67	120.80 ± 0.00	6.84 ± 0.00
F6	13.14 ± 2.65	0.89	0.12 ± 0.03	19.87 ± 4.90	120.50 ± 0.00	6.73 ± 0.00

SOR: surfactant-to-oil ratio. PDI: polydispersity index. Values represent mean ± SD.

**Table 3:** Minimum inhibition concentration (MIC) and minimum bactericidal concentration (MBC) of the nanoemulsions and *B. dracunculifolia* essential oil.

Microorganisms	Nanoemulsions	Essential Oil	Nanoemulsions	Essential Oil
	MIC (mg/mL)	MIC (mg/mL)	MBC (mg/mL)	MBC (mg/mL)
*S. aureus* ATCC 25923	7.5 ^a^	10.0 ^a^	10.0 ^a^	20.0 ^b^
*B. cereus* ATCC 14579	2.5 ^a^	10.0 ^b^	20.0 ^c^	15.0 ^d^
*S. mutans* ATCC 25175	2.5 ^a^	10.0 ^b^	10.0 ^b^	20.0 ^c^
*E. faecalis* ATCC 29212	7.5 ^a^	15.0 ^b^	20.0 ^c^	20.0 ^c^
*P. aeruginosa* ATCC 27853	5.0 ^a^	10.0 ^b^	>20	20.0 ^c^
*K. pneumoniae* ATCC BAA-1706	5.0 ^a^	15.0 ^b^	>20	20.0 ^c^
*S. enterica* ATCC 14028	2.5 ^a^	10.0 ^b^	>20	15.0 ^c^
*E. coli* ATCC 25922	5.0 ^a^	10.0 ^b^	>20	15.0 ^c^

Different letters within the same line mean significant differences at *p* ˂ 0.05 according to the Tukey test at the 95% confidence level.

**Table 4:** Antimicrobial activity of *B. dracunculifolia* essential oil and its nanoemulsions using the disk diffusion method.

Microorganisms	Zone Diameter (mm)	
	Nanoemulsions	Essential Oil
*S. aureus* ATCC 25923	18.5 ± 0.21 ^a^	19.4 ± 0.57 ^a^
*B. cereus* ATCC 14579	19.5 ± 0.21 ^a^	21.0 ± 0.11 ^a^
*S. mutans* ATCC 25175	20.0 ± 0.14 ^a^	23.0 ± 0.10 ^b^
*E. faecalis* ATCC 29212	20.0 ± 0.16 ^a^	20.7 ± 0.57 ^a^
*P. aeruginosa* ATCC 27853	n	n
*K. pneumoniae* ATCC BAA-1706	n	n
*S. enterica* ATCC 14028	n	n
*E. coli* ATCC 25922	n	n

Different letters within the same line mean significant differences at *p* ˂ 0.05 according to the Tukey test at the 95% confidence level; n = no inhibition zone.

**Table 5:** Composition of nanoemulsions from *B. dracunculifolia* essential oil.

Composition (%)	Nanoemulsions					
	1	2	3	4	5	6
Water	60.0	65.0	70.0	72.5	75.0	77.5
Oil	20.0	15.0	10.0	7.5	5.0	2.5
Tween 80	20.0	20.0	20.0	20.0	20.0	20.0

## Data Availability

The data presented in this study are available on request from the corresponding author. The data are not publicly available due to privacy interests.

## Supplementary Materials

The following supporting information can be downloaded at: https://www.mdpi.com/article/10.3390/antibiotics12121677/s1, Figure S1: Nanoemulsions from *B. dracunculifolia* essential oil, Figure S2: MBC of 10 mg/mL for *B. cereus* ATCC 14579 of *B. dracunculifolia* essential oil: 10 mg/mL (plates without bacteria); 7.5 mg/mL (plaques with bacteria); (C+) = positive control and (C−) = negative control, Figure S3: MIC of 7.5 mg/mL for *S. aureus* ATCC 25175 of nanoemulsions from *B. dracunculifolia* essential oil: 15 mg/mL (blue color), 10 mg/mL (blue color), 7.5 mg/mL (blue color), 5 mg/mL (pink color), 2.5 mg/mL (pink color), Figure S4: Results of disc diffusion method of *B. dracunculifolia* essential oil for: *S. aureus* ATCC 25923, *B. cereus* ATCC 14579, *S. mutans* ATCC 25175, *P. aeruginosa* ATCC 27853, *E. coli* ATCC 25922 and *K. pneumoniae* ATCC BAA-1706, Figure S5: Results of disc diffusion method of nanoemulsions from *B. dracunculifolia* essential oil for: *S. aureus* ATCC 25923 and *B. cereus* ATCC 14579.
