# Supplementary material for: Characterization and Determination of the Antibacterial Activity of Baccharis dracunculifolia Essential-Oil Nanoemulsions
Source: Antibiotics (Basel). 2023 Nov 29;12(12):1677. doi: 10.3390/antibiotics12121677 (PMC10740613; doi:10.3390/antibiotics12121677)
Supplement: Supplementary file 1 [file antibiotics-12-01677-s001.zip › antibiotics-2719163-supplementary.pdf]

antibiotics

Supplementary Materials

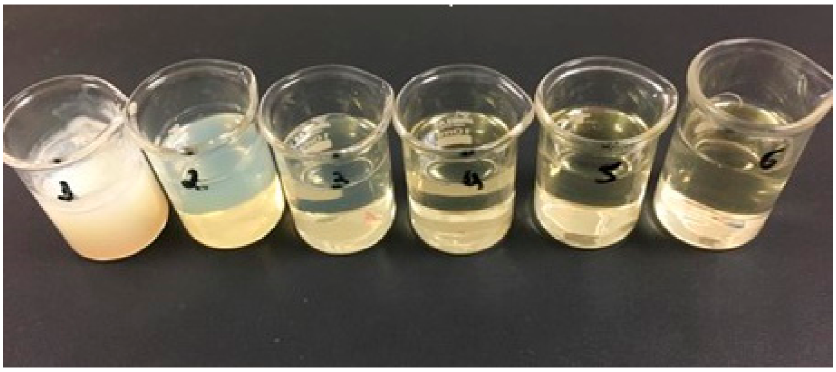

Figure S1. Nanoemulsions from *B. dracunculifolia* essential oil.

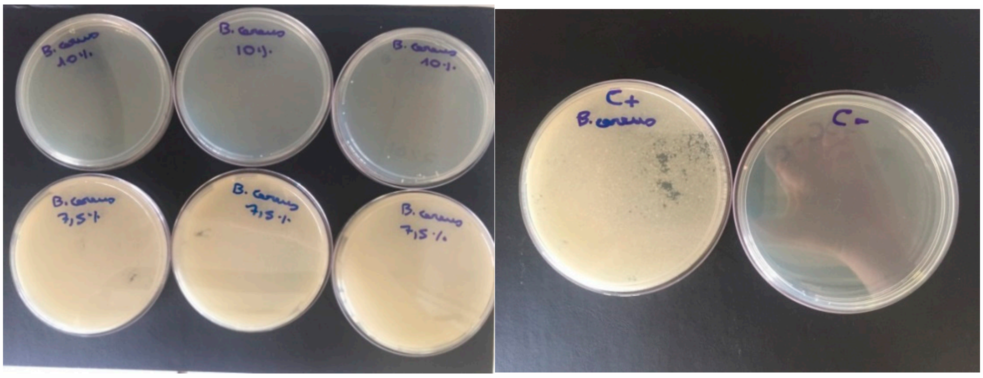

Figure S2. MBC (minimum bactericidal concentration) of 10 mg/mL for *B. cereus* ATCC 14579 of *B. dracunculifolia* essential oil: 10 mg/mL (plates without bacteria); 7.5 mg/mL (plaques with bacteria); (C+) = positive control and (C−) = negative control.

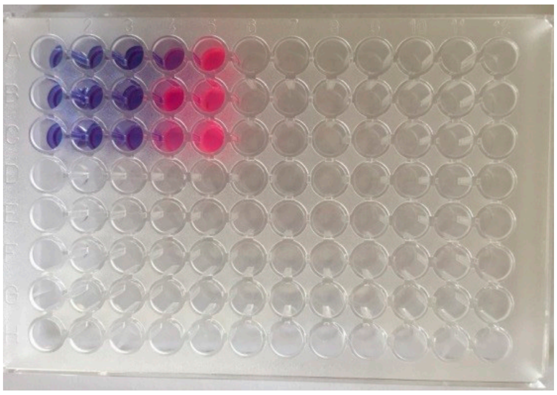

Figure S3. MIC (minimum inhibition concentration)of 7.5 mg/mL for *S. aureus* ATCC 25175 of nanoemulsions from *B. dracunculifolia* essential oil: 15 mg/mL (blue color), 10 mg/mL (blue color), 7.5 mg/mL (blue color), 5 mg/mL (pink color), 2.5 mg/mL (pink color).

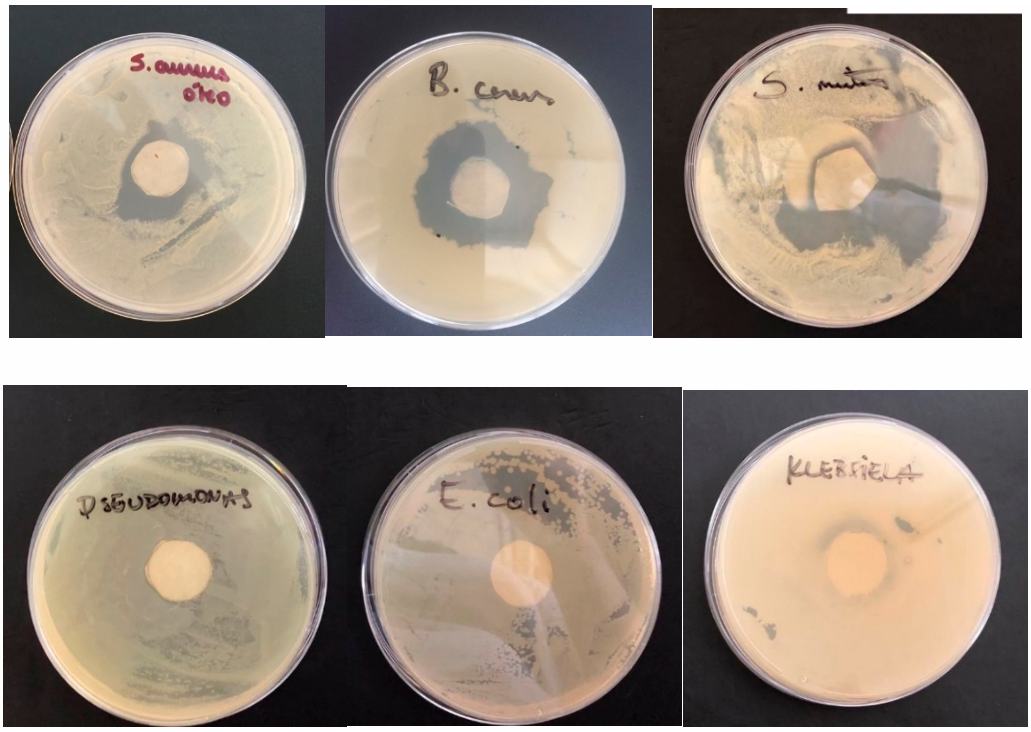

Figure S4. Results of disc diffusion method of *B. dracunculifolia* essential oil for: *S. aureus* ATCC 25923, *B. cereus* ATCC 14579, *S. mutans* ATCC 25175, *P. aeruginosa* ATCC 27853, *E. coli* ATCC 25922 and *K. pneumoniae* ATCC BAA-1706.

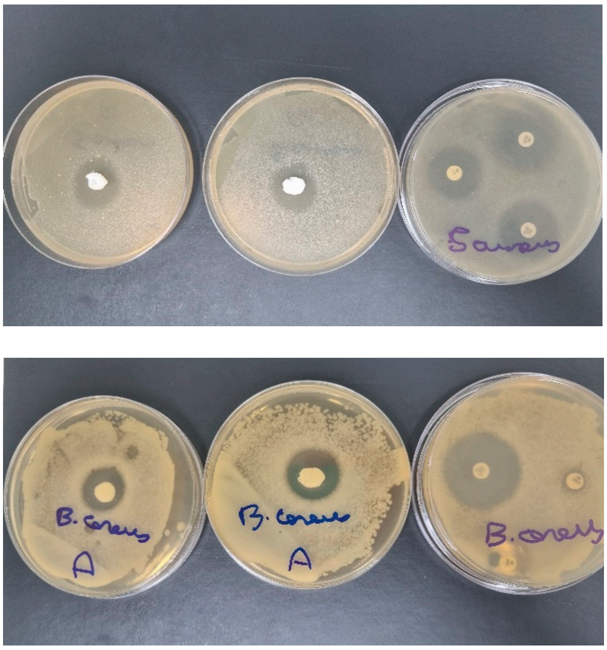

Figure S5. Results of disc diffusion method of nanoemulsions from *B. dracunculifolia* essential oil for: *S. aureus* ATCC 25923 and *B. cereus* ATCC 14579.
